# The effects of psychosocial interventions on reducing hospitalisation in people with dementia: an umbrella review

**DOI:** 10.1002/alz70858_100202

**Published:** 2025-12-25

**Authors:** Connie Howard, Andrew Sommerlad, Gill Livingston, Alba Fernandez‐Sanles

**Affiliations:** ^1^ Division of Psychiatry, University College London, London, London, United Kingdom; ^2^ Camden and Islington NHS Foundation Trust, London, London, United Kingdom; ^3^ University College London, London, London, United Kingdom; ^4^ Division of Psychiatry, University College London, London, Other, United Kingdom

## Abstract

**Background:**

People with dementia have a 42% increased risk of admission to acute hospitals compared to individuals without dementia, after accounting for their higher rate of physical comorbidities. Hospital admission is potentially harmful with a higher risk of falls, functional and cognitive decline, emotional distress and other hospital‐related adverse events. Moreover, admissions are costly, and a projected 1 in 4 hospital beds will be occupied by people with dementia by 2040. We, therefore, aimed to conduct an umbrella review to collate the current systematic review and meta‐analysis evidence on the effectiveness of non‐pharmacological interventions to reduce hospitalisation in people with dementia.

**Method:**

We registered the review and searched four databases (MEDLINE, Embase, Cochrane, CINAHL) until November 2024. We included peer‐reviewed systematic reviews which were: 1) studies of people with dementia and/ or their family carers or healthcare staff, 2) any psychosocial intervention regardless of setting, 3) comparison group as treatment as usual or evaluated before and after the intervention and, 4) outcomes related to hospitalisation such as hospital admission, readmission, delayed discharge, length of stay or emergency department visits. We assessed study quality with the AMSTAR II tool.

**Result:**

1,318 unique titles and abstracts were screened, of which 20 articles met inclusion criteria. These reviews comprised 97 studies involving 12,132 people with dementia in 17 countries. We will present evidence for all interventions, including environmental changes, exercise, case management, multidisciplinary discharge teams, and psychoeducation. Preliminary results suggest that there is limited and inconsistent evidence for specific psychosocial interventions to reduce hospitalisation in people with dementia. Many reviewers report significant heterogeneity between intervention types meaning meta‐analyses are rare.

**Conclusion:**

In this umbrella review, we will identify and classify promising non‐pharmacological interventions to reduce hospitalisation in people with dementia, guiding future research and implementation with the potential to reduce the individual and societal burden of acute care.

